# Author's reply

**Published:** 2010

**Authors:** R. Vimalavathini, S. M. Agarwal, B. Gitanjali

**Affiliations:** Department of Pharmacology, Jawaharlal Institute of Post Graduate Medical Education and Research (JIPMER), Pondicherry - 605 006, India

Dear Sir,

We fully agree with the view expressed by Dr. Abdus Salam[1] on our article.[2] However, we included communication-related factors within the category of patient-related factors and not as a separate entity. The fact that the patients' knowledge and attitude improved (in our study), showed that the intervention and our communication was effective. However, they were unable to comply with, and practice what they knew, because of the reasons we had outlined in the study. Adherence is a rather complex phenomenon, especially in chronic diseases, and to attribute poor adherence only to poor communication is trying to simplify a rather complex issue.
